# Prenatal diagnosis of a de novo 15q11.2 microdeletion in a maternal inv(4)(p15q31) fetus with increased nuchal translucency: A case report and literature review

**DOI:** 10.1097/MD.0000000000022496

**Published:** 2020-10-02

**Authors:** Meiling Sun, Fagui Yue, Yang Yu, Leilei Li, Yuting Jiang, Hongguo Zhang, Ruizhi Liu

**Affiliations:** a Center for Reproductive Medicine and Center for Prenatal Diagnosis, First Hospital; b Jilin Engineering Research Center for Reproductive Medicine and Genetics, Jilin University, Changchun, Jilin, PR China.

**Keywords:** 15q11.2 microdeletion, chromosomal microarray analysis, prenatal diagnosis, ultrasound abnormalities

## Abstract

**Rationale:**

15q11.2 microdeletion syndrome is a relatively rare chromosomal abnormality with incomplete penetrance and phenotypic variability. The reports on prenatal ultrasound abnormalities of fetus with 15q11.2 microdeletion are rare.

**Patient concerns:**

A 30-year-old woman was referred for genetic counseling and prenatal diagnosis at 19 weeks of gestation because of increased nuchal translucency in prenatal ultrasound findings and a history of spontaneous abortion.

**Diagnoses:**

The cytogenetic analysis showed the karyotype of the fetus was 46,XY, inv(4)(p15q31) and chromosomal microarray analysis detected a 0.512 Mb deletion in 15q11.2 region. We recalled the parents to determine the origination of these chromosomal abnormalities.

**Interventions:**

The pregnant woman chose to continue the pregnancies and finally delivered a healthy male infant at 39 weeks.

**Outcomes:**

The fetus inherited the inv(4)(p15q31) from his mother while the deletion in 15q11.2 was identified as de novo. Given the normal phenotype of the mother, it was reasonable to assume that the maternal inherited inv(4) in the fetus would not increase the risk of his abnormal phenotype. However, the pathogenicity of the microdeletion in 15q11.2 for the infant is unknown and long-term follow-up of progeny should be paid more attention.

**Lessons:**

The combined application of traditional banding technique and molecular cytogenetic techniques can not only detect chromosomal structural abnormalities, but also identify the subchromosomal imbalances, which is beneficial to genetic counselling and would offer more guidance to prenatal diagnosis.

## 1 Introduction

Chromosomal microdeletions and microduplications have been associated with clinic manifestations, characterized by intellectual disability (ID), developmental delay (DD), autism spectrum disorders and/or multiple congenital anomalies.[^1^,^2^] These submicroscopic deletions and duplications, which are typically in the range of 100 to 300 kb, could be detected by chromosomal microarray analysis (CMA).^[3]^ The copy number variants (CNVs) detected by CMA are associated with diverse clinical insignificance when the critical genes or important regulatory regions are located in the duplicated/deleted region.^[4]^

At present, 15q microdeletions are considered to be correlated with a variety of clinical phenotypes. Common 15q deletions are classified as follows: 15q11.2, 15q13.2-q13.3, 15q13.3, 15q14, 15q21.1-q21.2, 15q24, 15q24.1, 15q24.3-q25.2, 15q26, and 15q26.1.^[5]^ In particular, 5 breakpoints (BP1-BP5) in the proximal long arm of chromosome 15 (15q11-q13) are noteworthy because they increased the occurrence of CNVs within this region.^[6]^ Chromosome 15q11.2 microdeletion syndrome (OMIM: 615656) is an autosomal dominant disorder with incomplete penetrance and phenotypic variability, spanning approximately 300 to 500 kb between BP1 and BP2.^[7]^ The clinical phenotypes are mainly characterized by psychomotor developmental, speech delay, autism spectrum disorder (ASD), attention deficit hyperactivity disorder (ADHD), and seizures. In addition, other abnormalities, such as mild, moderate, and severe neurodevelopmental symptoms and congenital heart disease, were also reported.[^7^,^8^]

Here, we delineated a prenatal case with 15q11.2 (BP1-BP2) microdeletion, presenting abnormal ultrasound findings using chromosomal microarray analysis (CMA). Meanwhile, we also made a review on prenatal cases involving similar 15q11.2 deletion with our case.

## 2 Case report

A 30-year-old woman was referred for genetic counseling and prenatal diagnosis at 19 weeks of gestation in the Center for Reproductive Medicine and Center for Prenatal Diagnosis of First Hospital of Jilin University. Nineteen weeks sonography findings indicated that increased nuchal translucency was detected in prenatal ultrasound. And the woman had a history of spontaneous abortion. There was no teratogenic exposure or infectious diseases during mother pregnancy. She and her husband claimed that they were nonconsanguineous and healthy, and no family histories of diabetes mellitus or congenital malformations were found. The study protocol was approved by the Ethics Committee of the First Hospital of Jilin University (No.2019–283), and the informed written consents were obtained from this couple for publication of this case report and accompanying images.

## 3 Material and methods

### 3.1 Cytogenetic analysis

Chromosome analysis was performed on G-band metaphases prepared from cultured amino fluid cells and peripheral blood cells according to standard protocols. Fifty metaphases were analyzed for all samples. We described the karyotype according to the International System for Human Cytogenetic Nomenclature (ISCN 2016).^[9]^

### 3.2 Chromosomal microarray analysis (CMA)

CMA was performed on 10 ml uncultured amino fluid cells and 5 ml peripheral blood cells according to the manufacturers protocol by CytoScan 750K array (Affymetrix, Santa Clara, CA). Thresholds for genome-wide screening were set at ≥200 kb for gains, ≥100 kb for losses. The genomic coordinates were based on the GRCh37/hg19 build of the human reference genome. The final results were analyzed using the DECIPHER, database of genomic variants (DGV), Online Mendelian Inheritance in Man (OMIM), and so on.^[10]^

## 4 Results

**Figure 1:**
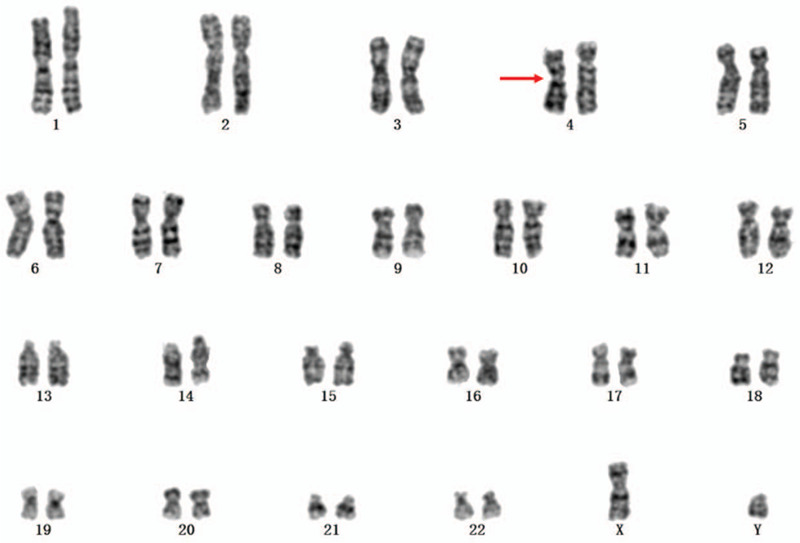
G-banding revealed the male infant with chromosomal karyotype 46,XY, inv(4)(p15q31).

**Figure 2:**
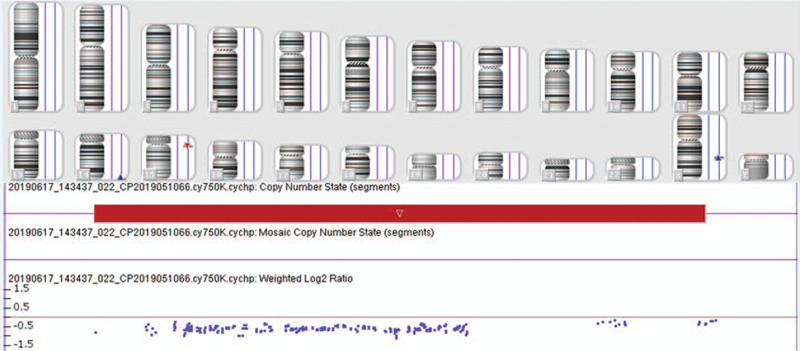
The result of CMA revealed a 0.512Mb deletion at 15q11.2, which was described as arr[GRCh37] 15q11.2(22770421-23282798)x1.

Chromosomal karyotypic analysis revealed the karyotype of the fetus was 46,XY, inv(4)(p15q31) (Fig. 1) and the result of CMA detected a 0.512 Mb microdeletion, shown as arr[GRCh37] 15q11.2(22770421–23282798)x1 (Fig. 2). We recalled the couple back for chromosomal karyotypic analysis and CMA verification. The husbands karyotype was 46,XY while the wifes was 46,XX, inv(4)(p15q31). CMA results of the couple were normal. So we concluded that the fetus got the inherited inv(4)(p15q31) from his mother, and 15q11.2 microdeletion in the fetus was de novo. Finally, the pregnant woman chose to continue the pregnancy and a healthy male infant was born at term. According to the follow-up outcome, the infants birth weight was 3300 g, and birth length was 50 cm and no apparent abnormalities were observed till now.

## 5 Discussion

We presented a rare prenatal case with a de novo 0.512 Mb microdeletion in 15q11.2, accompanied by increased nuchal translucency in prenatal ultrasound findings. Meanwhile, the fetus got inv(4)(p15q31) from his normal phenotypic mother.

In the proximal long arm of chromosome 15, a cluster of low copy DNA repeats located at the common 15q11-q13 breakpoints BP1-BP5, can frequently mediate various deletions and duplications via non-allelic homologous recombination.^[11]^ The typical deletion of 15q11-q13 can be classified as type I, involving breakpoints BP1 and BP3 (∼6Mb) and type II, involving BP2 and BP3 (∼5.3Mb).^[12]^ The 15q11.2 microdeletion which contains different regions between type I and type II deletion (BP1-BP2), is becoming an important research field in recent years. However, although 15q11.2 microdeletion is smaller than both type I and type II deletion, the typical features of 15q11.2 microdeletion carriers include neurobehavioral problems, developmental and language delays, intrauterine growth restriction and dysmorphic features.^[13]^

In a previous study with 200 patients with 15q11.2 microdeletion, developmental delay was the most common feature, accounting for 73% in the patients. Forty three percent individuals had abnormal brain imaging, and 26% had features of epilepsy.^[13]^ In another review of 56 patients with 15q11.2 microdeletion, 59% patients presented development delay, 36% patients presented speech delay. And among the patients beyond 1 year (49/56), 90% presented speech delay, 67% showed behavioral and neurological disorders such as ataxia, dyspraxia, hypotonia, obsessive-complusive disorder, and 25% showed seizures.^[14]^ All these studies suggested that CNVs in BP1-BP2 may increase susceptibility to neuropsychiatric or neurodevelopmental disorders. Nevertheless, some individuals with 15q11.2 microdeletion are clinically unaffected. To estimate the likelihood of 15q11.2 microdeletion causing dysmorphic features, Mohan et al^[15]^ calculated penetrance and obtained a value of 3.8%, suggesting low penetrance. Generally speaking, the 15q11.2 microdeletion is an apparent incomplete penetrance and variable expressivity.

To our best knowledge, the reports of prenatal abnormal ultrasound findings associated with chromosome 15q11.2 (BP1-BP2) microdeletion are rare. On one hand, this syndrome is not frequently associated with major structural abnormalities in fetal ultrasound. On the other hand, conventional cytogenetic analysis limits the detection of such microdeletion.^[14]^ To delineate the association between 15q11.2 microdeletion and ultrasound abnormalities in prenatal cases, we summarized the prenatal ultrasound findings in relevant cases sharing similar microdeletion region with our case in Table 1.[^7^,^16^,^17^,^18^,^19^] Among the 15q11.2 microdeletions, 4/10 cases were paternal inherited, 5/10 cases were maternal inherited, and only our case was de novo. The de novo 15q11.2 deletion was rare, which accounts for only 5% to 22% in reported carriers.^[20]^ The high incidence rate of clinical characteristics in these fetuses was as follows: increased nuchal translucency (4/10), intrauterine growth restriction (IUGR) (3/10), microcephaly (3/10), congenital heart disease (3/10). In addition, other malformations were also observed in fetuses, such as pulmonary atresia, exomphalos, micrognathia, bilateral cleft lip and palate, clubfeet. It seemed that prenatal cases with 15q11.2 (BP1-BP2) microdeletion may suffer the risk of microcephaly, IUGR, increased nuchal translucency and some heart-related diseases. The genotype-phenotype of 15q11.2 microdeletion in fetuses has remained elusive and more evidence is required to determine this association.

**Table 1:**
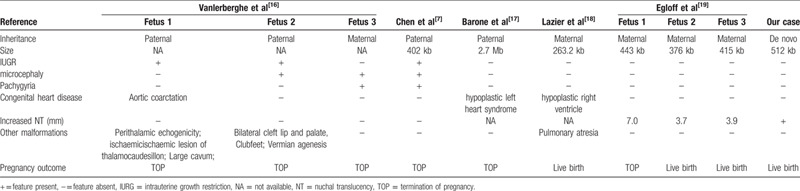
Clinical date of fetuses with the 15q11.2 (BP1-BP2) microdeletion in prenatal diagnosis.

According to the DECIPHER database, the critical region in chromosome 15q11.2 contains 4 non-imprinted genes of *NIPA1* (OMIM 608145), *NIPA2* (OMIM 608146), *CYFIP1* (OMIM 606322) and *TUBGCP5* (OMIM 608147), which are highly conserved and biallelically expressed, which play critical roles in brain development and function.[^7^,^14^] As the best-studied gene in this region, *NIPA1* is highly expressed in neuronal tissue, which is associated with autosomal dominant hereditary spastic paraplegia and mediates Mg^2+^ transport.^[21]^ The *NIPA2* gene encodes a protein that plays a role in Mg^2+^ transport in renal cells. Jiang et al^[22]^ summarized that the haploinsufficiency of *NIPA2* might be a candidate mechanism for child absence epilepsy/idiopathic generalized epilepsies phenotypes caused by 15q11.2 microdeletion.[^12^,^22^] Maver et al^[23]^ found that the loss of *TUBGCP5* due to 15q11.2 microdeletion might be involved in the development of microcephaly. The protein product of *CYFIP1* has been proved to interact with FMRP, the protein coded by the *FMR1* gene, which is responsible for the Fragile X syndrome. Fragile X syndrome now is the common cause of familial intellectual disability.^[24]^

Currently, comprehensive interpretation and counseling for 15q11.2 microdeletion region in prenatal samples remains challenging. This type of deletions is not easily detected in prenatal diagnosis because fetal ultrasound may not always infer significant structural abnormalities. Most children with such microdeletion can survive normally, but they may exhibit various degrees of physical or mental developmental abnormalities after birth.^[25]^ Due to the incomplete penetrance of 15q11.2 microdeletion carriers, long-term follow-up till adulthood for the healthy infant in our report is necessary.

Traditional cytogenetic techniques play critical roles in identifying chromosomal inversions and translocations which could not be detected by CMA.^[8]^ While CMA allows the detection of microscopic imbalances in the kilobase range due to high resolution.[^3^,^4^] The combined application of traditional banding technique and molecular cytogenetic techniques had complementary advantages, which could offer more details and information for genetic counseling. In addition, considering the wife's abnormal karyotype with inv(4), preimplantation genetic diagnosis (PGD) is an appropriate choice which could decrease risk of the spontaneous abortions if they intend to conceive again.

## 6 Conclusion

We analyzed a rare prenatal inv(4)(p15q31) case with de novo 0.512 Mb microdeletion in 15q11.2 region, accompanied by increased nuchal translucency. Our presentation not only highlights the correlation between 15q11.2 microdeletion and prenatal ultrasound abnormalities, but also emphasizes the necessity of long-term follow-up for 15q11.2 microdeletion carriers. Comprehensive interpretation and genetic counseling for 15q11.2 microdeletion remain challenging, and further studies should be gathered to obtain a better understanding of the impact of 15q11.2 microdeletion.

## Author contributions

**Conceptualization:** Leilei Li, Hongguo Zhang, Ruizhi Liu.

**Data curation:** Yang Yu.

**Formal analysis:** Yang Yu, Yuting Jiang.

**Funding acquisition:** Ruizhi Liu.

**Investigation:** Meiling Sun, Fagui Yue, Yuting Jiang, Ruizhi Liu.

**Methodology:** Yang Yu, Yuting Jiang, Hongguo Zhang.

**Project administration:** Ruizhi Liu.

**Supervision:** Fagui Yue, Leilei Li, Hongguo Zhang.

**Validation:** Fagui Yue, Hongguo Zhang, Ruizhi Liu.

**Writing – original draft:** Meiling Sun.

**Writing – review & editing:** Ruizhi Liu.
